# S14-2 Sports promotion in children and adolescents: analysis of good practice interventions in 7 countries

**DOI:** 10.1093/eurpub/ckad133.068

**Published:** 2023-09-11

**Authors:** Cécile Grobet, Renato Mattli, Linda Vinci, Maria Carlander, Sonja Kahlmeier

**Affiliations:** Zurich University of Applied Sciences, ZHAW, Switzerland; Zurich University of Applied Sciences, ZHAW, Switzerland; Zurich University of Applied Sciences, ZHAW, Switzerland; Zurich University of Applied Sciences, ZHAW, Switzerland; The Swiss Distance University of Applied Sciences, Switzerland

## Abstract

**Purpose:**

There are many interesting sports promotion interventions in European countries. Which ones are effective and can serve as good practice examples among children and adolescents? What should be considered when policies or programmes are transferred to other countries? This study has examined sports promotion interventions of seven European countries. It provides an overview of effective interventions and considers transferability to other countries.

**Project description:**

The Swiss Federal Office of Sports assigned a study to identify effective sport promotion interventions in seven selected European countries with a potential to be transferred to Switzerland. The seven countries were: Austria, Germany, England, Finland, France, The Netherlands, and Norway.

To identify the interventions, we conducted desk research and interviews with country representatives.

In total, we identified 73 sport-promoting policies, action plans or programmes in the seven countries. As part of the detailed analysis of the most promising interventions, we have examined the national strategy “On the move” of Finland and the national action plan “School sport and activity action plan” of England. These superordinate interventions stand out for their clear objectives and comprehensive implementation. In addition, we have identified several interventions for which we consider a high added value in terms of sports promotion and a feasible implementation in Switzerland or other countries. These are “The Daily Mile” from Germany (15 minutes run or walk every day at school), “Girls Active” from England (enhancing of sport and physical activity in girls in the school setting), “Doorstep Sportclub” from England and “Community Sport Coaches” from the Netherlands (both address in particular children and adolescents with a migration background or low socio-economic status and are offered through the municipality and the associations).

**Conclusions:**

This study gives an overview about effective sport promotion interventions targeting children and adolescents of seven European countries. Interventions that include comprehensive measures such as a national strategy or low-threshold programmes in schools or communities that address vulnerable groups such as girls, educationally disadvantaged families, children and youth with a migration background or low socio-economic status are particularly interesting from a Swiss perspective and certainly also for other European countries.

